# Taking Turns or Halving It All: Care Trajectories of Dual-Caring Couples

**DOI:** 10.1007/s10680-018-9473-5

**Published:** 2018-03-07

**Authors:** Helen Eriksson

**Affiliations:** Stockholm University Demography Unit, Department of Sociology, Stockholm University, 106 91 Stockholm, Sweden

**Keywords:** Child care, Dual-caring, Parental leave, Sweden, Sequence analysis

## Abstract

Interview and observational studies document that dual-caring is characterized by temporality. Two ‘ideal-typical’ trajectories are identified: ‘halving it all’ in which couples divide care equally on a daily or weekly basis and ‘taking turns’ in which parents take month- or year-long turns in serving as primary caregivers to the child. This study utilizes a new source of couple-level longitudinal information on parental leave to investigate dual-caring trajectories in contemporary Sweden. Results show that while care trajectories in which only one parent serves as the primary caregiver can be captured without longitudinal information, the dominant dual-caring trajectory cannot. In fact, despite a uniquely flexible parental leave system that allows egalitarian couples to share care on a daily basis, most couples do not share care in every point in time, but ‘take turns’ in serving as the primary caregiver to the child, with the mother’s ‘turn’ preceding the father’s. The results demonstrate that cross-sectional and aggregate measures of child care may fail to detect emerging trends in dual-caring.

## Introduction

Time inflexibility of child care is increasingly recognized for its importance in the creation of gender inequality within the couple; while other kinds of unpaid work can be ‘fit in’ around work schedules, young children need to be minded 24/7 (Bianchi et al. 2012, p. 60). Even couples with egalitarian division of labor before the transition to parenthood shift to a remarkably gendered division of labor after the birth; women substitute family work for paid work, while men’s time allocations, with few exceptions, remain rather static (Dribe and Stanfors 2009). Understanding the division of parental child care and, in particular, care that requires time away from paid work is therefore central to understanding reproduction of gender inequality in the family as well as in the labor market.

Couple arrangements in which both partners substantially allocate time from paid work to child care, what is termed ‘dual-caring,’ challenges and has the potential to alter the current gender system. We know very little about these arrangements. When both partners take time off work to care for a newborn child, who is at home when? A few interview and observational studies document that dual-caring is characterized by *temporality.* Two ‘ideal-typical’ trajectories are identified: ‘halving it all’ in which couples divide care equally also on a daily or weekly basis and ‘taking turns’ in which parents take turns to serve as primary caregivers to the child. While ‘halving it all’ would be captured as egalitarian caring in the cross section, ‘taking turns’ would be captured as a heavily skewed division of care in favor of the parent acting as primary caregiver at a given time. Longitudinal data including caregiving of both parents with short enough intervals of observation are required to capture the particular ‘turn-taking’ behavior documented in qualitative studies.

The aim of the current paper is twofold: First, I develop a temporal conceptualization of a ‘dual-caring trajectory’ as a trajectory of time allocations by mothers and fathers over the child’s early years. Second, I utilize a new source of couple-level longitudinal information on parents’ caregiving to investigate dual-caring trajectories in contemporary Sweden. Sequence analysis is applied to identify clusters of ideal-typical trajectories. The validity of the care trajectory as a temporal measure of care is explored by examining bivariate associations of cluster membership by educational level, income and employment status of the mother and the father. The Swedish case is particularly well suited for understanding the ways in which couples may overcome barriers to dual-caring. Substantial numbers of Swedish fathers allocate time from paid work to child care, and the Swedish parental leave system allows for care trajectories ranging from ‘halving it all’ on a daily or weekly basis to ‘taking turns’ of around 6 months to 1 year each.

## Conceptualizing and Measuring Dual-Caring

Child care demands are the primary consideration in shaping a couple’s division of labor. The birth of a child induces a remarkably gendered division of labor, even in pre-birth egalitarian couples (Dribe and Stanfors 2009). Dual-caring, as in couple arrangements in which both partners substantially allocate time from paid work to child care, has the potential to alter the current gender system. A correct conceptualization and measurement of dual-caring is therefore essential for understanding the determinants and consequences of gendered divisions of labor.

Many couples striving for dual-caring describe themselves as ‘halving it all,’ i.e., maintaining a 50–50 division of child care and family work at all times. In Deutsch’s (1999) extensive interview study of household strategies, egalitarian couples were those that ‘overall, when everything that went into the care of children in a typical week was taken into account, the work was split 50–50’ (Deutsch 1999, p. 5). This arrangement, according to Deutsch’s observations, leads fathers to be on a par with the mothers and ‘primary in their children’s lives’ (Deutsch 1999). Such couples would be identified as egalitarian in their care of children whether they were observed at one point in time or over a longer period of time.

Equal engagement as early as possible, especially through father’s ‘early infant care’ (Coltrane 1996), is key to this approach. Arguments for the importance of the period immediately following the birth of the child are made in a number of studies throughout the Anglo-Saxon context. In the USA, fathers not participating in child care immediately might be ‘discouraged from those tasks because mothers spend more time with the child after the birth and, hence, become the experts on that child’s care’ (Nepomnyaschy and Waldfogel 2007, p. 429). In Canada leave-taking together immediately after the birth of the child is expressed by parents as a way to ‘support each other and work together in learning to care for the child’ (McKay and Doucet 2010, p. 310), and it is argued that such structurally similar transitions into parenthood will make parents develop similar understandings and enactments of parenting (Rehel 2014, p. 111).

These studies generally show that early engagement is positively associated but not equivalent to long-term engagement. As shown by Miller (2011) in a longitudinal interview study of first-time fathers in the UK, dual-caring in the early weeks following the birth is not sustained as fathers re-engage in full-time paid work. She documents a ‘naivety’ about practical management of dual-caring when the fathers work full-time and mothers are on leave full-time, thereby suggesting that a dual-caring strategy requires an absence from paid work for both parents.

Empirical studies from the European and especially Nordic context document an alternative strategy of ‘taking turns.’ As almost all parental caregiving in the first years of the child’s life that requires an absence from paid work goes through the parental leave systems in these countries, studies on gender equal parenting are often found in the extensive literature on the division of parental leave. In comparison with the distinction made in time-use surveys between caring activities performed alone or with the mother present (Craig and Mullan 2011; Raley et al. 2012), these studies define extended solo-caring as monthly periods of daylong care during which the other parent is at the workplace (cf. Wall 2014). In an interview study of pre- and post-birth middle-class couples in Sweden, Roman (2014) reports how egalitarian mothers and fathers seek to protect both the mother’s first leave period as a ‘matter-of-course’ and the father’s ‘right’ to take over later on.

‘Taking turns’ in managing all tasks and full parental responsibility are important in this respect; egalitarian parents describe how they aim at a situation in which ‘he was the one who knew and she was the one who had to ask him’ (Roman 2014, p. 76). The absence of the mother is part of an explicit strategy articulated by both mothers and fathers to counteract the ‘manager-helper dynamics’ that may arise when one parent spends disproportionally more time with the child (Coltrane 1996; Rehel 2014). Finnish (Eerola 2014), Norwegian (Stefansen and Farstad 2010) and Icelandic (Farstad 2014) fathers and mothers practicing extended solo-caring emphasized ‘private time’ with the child for making fathers’ caregiving responsibilities ‘equal with those of the mother, unconstrained by gender’ (Eerola 2014, p. 316). Inequality at different points in time is thus identified as a means to achieve dual primary caregiving.

The characteristic inconsistency between making discursively legitimate claims of equal sharing and realizing unequal practices (Ferree 2010, p. 427) may play out somewhat differently when adopting a turn-taking strategy. Rather than falling back into secondary caregiving as documented among UK fathers by Miller (2011), Swedish egalitarian parents adapt to gendered workplace and economic ‘circumstances’ (Roman and Peterson 2011, p. 104) by realizing a care period *long enough* for the fathers to ‘take over’ care (Almqvist and Duvander 2014; Alsarve and Boye 2012; Roman 2014; Roman and Peterson 2011). Long enough has been suggested by Swedish and Icelandic parents as around 3 months of extended solo-caring (Farstad 2014; Roman 2014).

Current measures of care capture only sharing that includes no elements of turn-taking. Time diaries as the gold standard for quantitative evidence on division of child care time refer to 24-h periods only. Although these studies have provided ample evidence of differentials in time inequalities (Craig and Mullan 2011; Dribe and Stanfors 2009; Sayer et al. 2004), they cannot correctly capture turn-taking as described above. Studies on division of paid parental leave gathered either from surveys or from administrative data have documented most of what we know of inequalities in time allocations from paid work to child care. The typical measure of care sharing in these studies, annual uptake of parental leave (Duvander and Johansson 2012; Gíslason and Eydal 2011; Sundström and Duvander 2002), is, however, too crude a measure to capture temporality in care sharing.

## Opportunities and Constraints for Dual-Caregiving in the Swedish Context

For parents with children under 2 years old the most important parts of the Swedish welfare system are parental leave and public childcare. Paid parental leave is used for virtually all children (99.7%, own calculations), typically for a period of between one and 2 years (Swedish Social Insurance Agency 2011a), and most children are enrolled in publicly subsidized child care at the end of this period (Swedish National Agency for Education 2007). In 2009, the year in which children included in this study were born, parents were entitled to 390^1^ income-related job-protected parental leave days. Just under 80% of earnings up to a cap of 428,000 SEK (about 61,000 USD) were replaced. In 2011 about 10% of mothers and 25% of fathers were estimated to have earnings that were higher than the cap (SSIA 2011b). Many employers, however, provided benefits that cover 80–90% of earnings above the cap for a limited period of time (Sjögren Lindquist and Wadensjö 2005). Parents without prior earnings received a low flat rate (180 SEK or 26 USD per day). Of all parents claiming benefits in 2009, around 6% of mothers and 2% of fathers were paid on the flat rate because of not having prior earnings (SSIA 2011a). Another 90 days could be paid at the flat rate for all parents.

Even compared to the other Nordic countries, the Swedish parental leave system has been highly flexible from the start (Rostgaard 2002); parents could shift caregiving even by the hour. The primary restriction was that 60 days of the total available paid leave were reserved for each parent (the ‘daddy months’). Parents could also claim unpaid job-protected leave during the first 18 months. By accepting a replacement rate of less than the full 80% of earnings, unpaid leave could be used to extend the length of job-protected parental leave from about 1 year to a little more than 2 years.^2^ A typical arrangement for the first 2 years is to be on leave full-time, but claim only 5 out of 7 days per week, thereby receiving benefits that amount to 5/7 of the full 80% of earnings (Duvander and Viklund 2014). Mothers and fathers seem to extend their leave to the same extent (Duvander and Viklund 2014). Paid leave days could be used until the child turned 8 years old. Parents also had the right to work reduced hours (75% of normal hours) during these 8 years (SSIA 2011a).

Publicly subsidized child care was universally provided to all children 12 months and older. Most children were therefore enrolled when the parental leave period ended in the child’s second year of life (Evertsson and Duvander 2011). Since 2002 also children of unemployed parents or parents on parental leave may be enrolled (SNAE 2007). In 2011 88% of all 2-year-olds were enrolled in public child care (SNAE 2015). The cost of public child care is heavily subsidized; starting in 2002 parental fees for the first child are capped at 3% of the household income or a maximum of 1260 SEK (~ 200 USD) per month covering only about a tenth of the costs (SNAE 2007). Other types of informal care are rare; in a typical week, only 1.5% of 0–2-year-olds are estimated to be in any informal care such as with grandparents or nannies, compared to the OECD average of 24% (OECD 2015). Sweden almost entirely lacks the low-skilled and low-paid child care workforce of the USA (Morgan 2005). Labor market regulations, a compressed wage structure and high rates of unionization all contribute to a high cost of labor and thereby a low affordability in buying these services (Morgan 2005).

Parents’ use of flexible parental leave and subsidized child care is shaped by Sweden’s political and cultural context. Policy-making surrounding father care has a long tradition in Sweden, and the Swedish case has been described as the most institutionalized shift of fathers’ role from provider to caregiver (Bergman and Hobson 2002). Reformulating the idea of gender specificity and women’s rights into an idea of gender sameness and gender equality made possible the 1974 introduction of parental, as opposed to maternal, leave (Cedstrand 2011). Many studies have described the emergence of the child-centered fatherhood norm in Sweden, in which ‘the involved father’ takes on all practical aspects of caring for the children (Almqvist 2008; Johansson and Klinth 2008; Plantin 2007). Indeed, Sweden in the 2000s provided the first context in which evidence was found of similarity in the effects of parenthood on time use for men and women (Dribe and Stanfors 2009). Swedish women have been virtually on a par with men in labor force participation at least since the mid-1980s (Lewis and Åström 1992).

Much gender inequality remains, however. For example, fathers used only 22% of all paid days in 2009 (SSIA 2011a). Job and career costs of leave-taking are higher for men than for women, presumably because men are expected to take leave to much less extent than women (Albrecht et al. 1999; Evertsson and Duvander 2011). Taking the 2 months reserved for fathers may not be viewed by employers as a signal of low work commitment, given that the leave cannot be transferred to the mother (Brandth and Kvande 2009), but the longer leaves that would be required for full dual-caregiving remain a potential signal. The high levels of gender segregation by occupation, sector and workplace in Sweden (Statistics Sweden 2009) may further accentuate the gender differences in signaling costs of leave. The gender wage gap is also still substantial. In 2008, the weighted value of women’s earnings (taking account of age, educational background, full-time/part-time work, sector and educational group) was 92% as that of men (Statistics Sweden 2010). The wage gap in couples is further accentuated by assortative mating, for example in how men are on average 2 years older than women in couples that have their first child (Kolk 2015).

## Data

This study uses register data from the Swedish Social Insurance Agency (SSIA), the government agency administering the parental leave system. The parental leave system effectively captures care that requires time off paid work for the entire parental population. Data are recorded for every claim each parent makes and are thus not subject to measurement errors or memory lapses as would be the case for survey reports over such an extended period.

The SSIA data are comprised of dated episodes in which either parent claimed paid parental leave; for this study, episodes from birth up until the child is 2 years old are used.^3^ This information comes directly from parents’ claims on parental leave and is the most detailed information on parental leave usage available in Sweden. Parents are free to construct episodes of leave in any way they want. Episodes may consist of only 1 day and as little as an eighth of the day (1 h). As previously described, parents may choose any amount of days within an episode to remain unpaid or they may choose to claim episodes with gaps between them. Swedish registers contain only annual income data, i.e., no monthly earnings or days worked for pay, so it is not possible to distinguish parents with earnings during a particular day or week or month.

During the first 2 years after the first birth, 87,219 episodes of parental leave were reported, about 40 episodes per child. Each episode is connected to the ID number of the child for which the days are taken and the ID number of the claimant parent. For each episode, the start and end dates and the number of days the parent claimed within that particular period are provided. Episodes thus consist of a given number of days between two calendar dates for which payments are requested. The data contain no information on the exact days of the week for which paid leave was used.

Although the parent claiming leave is by law required to care for the child in the given day, it is of course impossible to monitor parents’ activities on the days they take leave. Regular checks with workplaces and public child care facilities for parents’ claiming temporary leave for care of sick children find very low levels of fraud (Häkkinen Skans and Johansson 2014). It is certainly possible for parents to take leave when the other parent is not working (e.g., weekends or holidays) and is also available to care, but having data for both parents and setting a relatively high threshold for periods of solo-leave-taking increases the likelihood that primary caregiving is provided by the parent on leave. Perhaps most important for a range of labor market consequences of leave, these data accurately capture if and when mothers and fathers were absent from paid work to care for their children.

The population under study is a 5% random sample of all different-sex couples registered in Sweden and having their first child in 2009: 2158 children and 4316 parents.^4^ Parental couples are connected via each parent’s biological link to the child, identified in Statistics Sweden’s Multi-generation Register (Statistics Sweden 2008). Adoptive parents are excluded as no adoption dates are provided in the data, and parents with multiple births are excluded as parental leave regulations are different than to those of single births. Total exclusions amount to about 1.6% of all first-born children born in Sweden in 2009. Separated parents are not excluded because parental leave eligibility depends only on legal custody and not on co-residence. In 2011, about 96% of all Swedish 2-year-olds were in joint custody (Statistics Sweden 2013).

For the bivariate associations, administrative register data connected to the episode information are used to identify educational level, income and employment status of the mother and the father. Educational level is derived from the Swedish ‘SUN2000’ coding scheme (equivalent to the International Standard Classification of Education, ISCED 97) and categorized as: *Primary* (maximum 9 years of schooling), *Secondary* (post-secondary schooling, 10–12 years) and *Tertiary* (college education of at least 3 years). Income is measured as declared annual income to the Swedish Tax Agency in year 2007.^5^ Employment status is derived from the ‘RAMS’ register maintained by Statistics Sweden into categories: *Unskilled employment* (below the third category of SSYK 96, the Swedish equivalent to the international ISCO-88 classifications), *Skilled employment* (third category and above), *Self*-*employment* (income registered from own firm) and *Not in employment* (no registered employment). For information on included registers, see Statistics Sweden 2016.

## Methods

To extract meaningful patterns from the temporally very detailed data, sequences of monthly uptake of paid leave are created from the episode information. The final result is a sequence of data points containing the number of paid leave days used by each parent for months 1–24 of the child’s life. If the episode falls within a given month of the child’s life, the total days claimed are assigned to that month; if the episode stretches over the turn of the month, days of leave are allocated to each month of the child’s life in proportion to the number of days in the episode that fall in each adjacent month. Minor misclassifications of single days may occur when parents have claimed only parts of weeks that stretch over the turn of the month.

Both parents’ monthly paid days of leave are used to classify the couple’s caregiving in a given month as only the mother, only the father, neither or both parents. A parent is classified as the only primary caregiver if she/he is the only one using paid days in a given month. Both are classified as primary caregivers if they both use paid leave in a given month.^6^ Sequence and cluster analyses were carried out using different minimum thresholds for primary care, ranging from any day to 20 days. An artificial increase in non-parental care months was observed using thresholds of more than 5 days per month, especially in the first year when parents were in most cases the only possible caregivers of the child (see discussion above). This finding is consistent with the known practice of using vacation days, both saved and granted during parental leave, in place of parental leave days during the child’s first year (Alsarve and Boye 2012; Sjögren Lindquist and Wadensjö 2005). As the point in time in which vacation days are used is of no theoretical importance for the construction of care trajectories, a 5-day limit was therefore chosen. Results were essentially the same with higher thresholds up to 20 days per month.

The care trajectory is therefore represented by the sequence of monthly care arrangements classified as mother only, father only, both or neither taking at least 5 days of paid leave. Sequence analysis is applied to the 24-point sequences to capture a holistic perspective on the care trajectory including concepts such as *timing* of mother care, father care and non-parental care, *duration* of each parent’s periods of primary care and *sequencing* in how parents may transfer caregiving between each other.

Sequences are analyzed using optimal matching (OM) analysis, the most broadly used sequence analysis technique in the social sciences (Aisenbrey and Fasang 2010; for overview, see Blanchard et al. 2014; MacIndoe and Abbott 2004). OM compares each sequence to any other sequence in the data. In each comparison, OM algorithms are applied to calculate a distance measure reflecting the similarity between the pair of sequences. Distances are measured in terms of elementary operations required to turn one sequence into another. The basic transformations include substituting, inserting or deleting an element of the sequence, and each of these transformations carries a specified cost (MacIndoe and Abbott 2004). The output of OM is a matrix of distances between each pair of sequences in the data. The particular OM technique used here is dynamic Hamming matching (DHM), introduced by Lesnard (2010) to capture the temporal dimension of sequences. By using only substitution costs and letting these vary with time by transitions between states, DHM is designed to identify socio-temporal patterns in sequences (Lesnard 2010). In the study of care trajectories, the temporal dimension of care is crucial, i.e., it is not just the occurrence of care that is of interest, but also the point in the child’s life at which, for example, fathers enter as primary caregivers.

The values of the OM matrix are analyzed by means of cluster analysis to generate clusters of more or less homogenous groups of sequences. The cluster technique used here is Ward’s algorithm (Ward 1963). There are no established cluster cutoff criteria widely applied to sequence analysis, and most statistical criteria are not easily transferable to sequential data (Aisenbrey and Fasang 2010). “Appendix” provides cluster cutoff criteria transferred by Studer (2013) to the OM dissimilarity matrix. Figure 2 provides a graphical display of measure values for cluster solutions between 1 and 20 possible clusters, and Table 3 provides measure values for the four highest local maxima. Both measures suggest substantial heterogeneity in care arrangements. The point biserial correlation, calculated as the correlation between each pairwise dissimilarity measure and a corresponding binary value for belonging to the same cluster or not (Milligan and Cooper 1985), reaches its maximum at a 17-cluster solution. The average silhouette width, comparing the distance of each observation to members of the same cluster with those of the nearest cluster (Rousseeuw 1987), suggests two local maxima: a 2-cluster solution and a 16-cluster solution. Because the 2-cluster solution distinguishes only between those care arrangements that include only mother care versus all other care arrangements (not shown here), the 17-cluster solution was chosen as a better representation of the theoretical conceptualization of temporal care. The R packages TraMineR (Gabadinho et al. 2010) and WeightedCluster (Studer 2013) are used for all analyses.

## Results

**Fig. 1:**
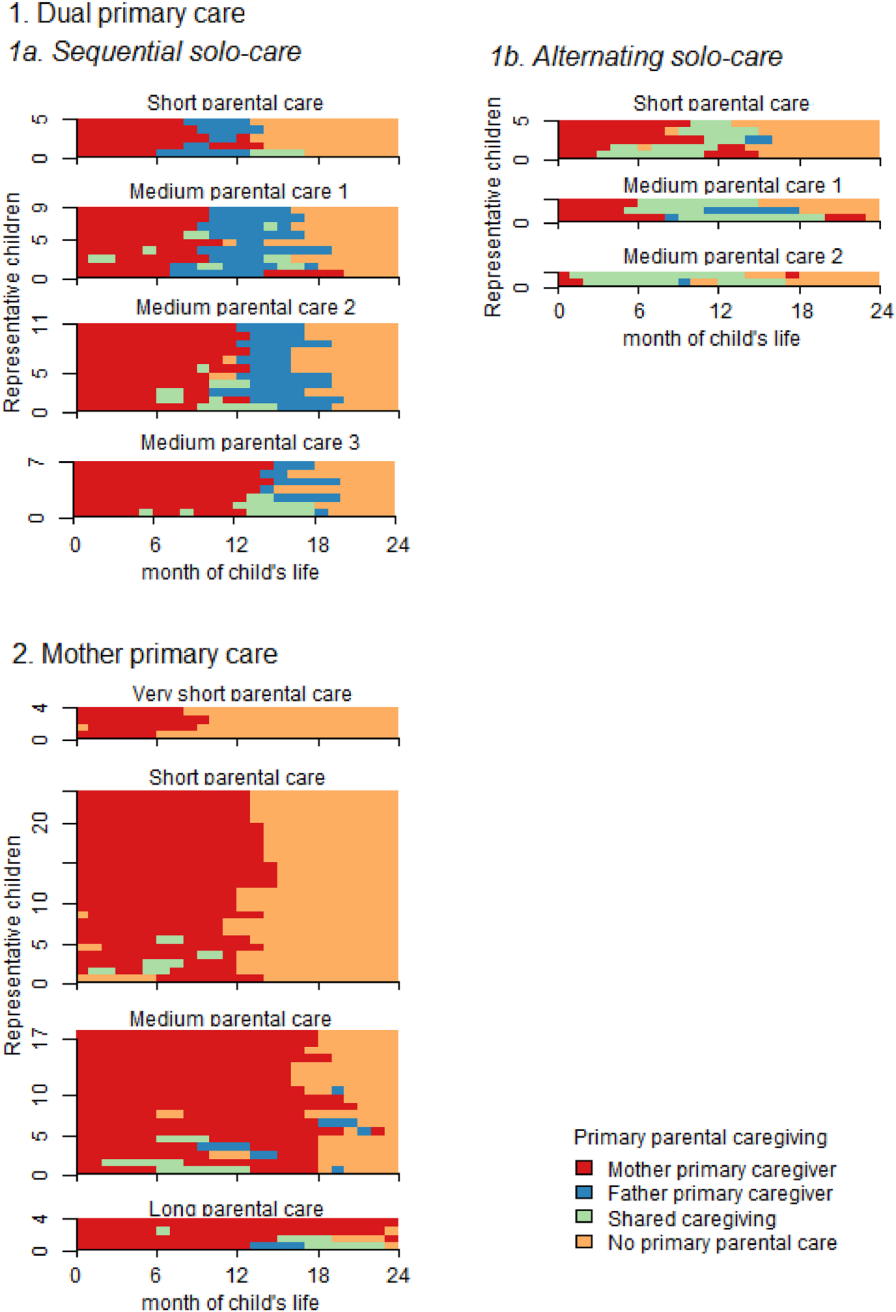
Relative frequency sequence plots of care trajectory clusters as generated through sequence and cluster analyses. Each individual child represents a 1% group of the sample. Sequences within each cluster are sorted in ascending order by how similar the sequence is to the cluster medoid

**Fig. 2:**
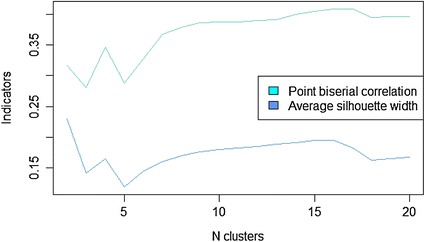
Average silhouette width and point biserial correlation for cluster solutions 1–20 (Studer 2013)

Figure 1 provides a visual representation for 11 of the 17 clusters of care trajectories. The remaining six clusters were all clear and homogenous but represented only marginal phenomena and were therefore not included in the figure. State distribution plots for all 17 clusters are provided in “Appendix.” In each panel, horizontal bars represent monthly primary caring of a number of representative children in each cluster. The colors represent different combinations of parental or non-parental care: mother only (red), father only (blue), both (green) and neither (orange). The height of the figure represents the percentage of couples in the cluster. Plotting all sequences in the cluster would make the figures unreadable (Fasang and Liao 2014), so Fig. 1 shows a selection of representative children, each representing 1% of the entire sample. For example, 5% of Swedish couples use the care trajectory of the top-left cluster in Fig. 1, so the strategy is represented by five horizontal bars with slightly different care trajectories. Each one-percent group is represented by the medoid (Fasang and Liao 2014), analogous to a mean, i.e., the specific sequence with the least distance to all other sequences in the group (Aassve et al. 2007). Sequences within each cluster are sorted in ascending order by how similar the sequences are to the cluster medoid. This sorting principle ensures that heterogeneity within each cluster is brought out and visualized in ascending order within each cluster panel. The first child in the first left-hand cluster experienced solo-mother care for the first 8 months of life, solo-father care for the following 5 months, and no parental care for the remaining 11 months until age two. Because public childcare is universally available from the time the child is 12 months, non-parental care is most likely provided by a preschool.

Clusters are sorted in Fig. 1 along the three main dimensions that notably distinguish them from each other. First, in terms of primary caregiving, two large and distinct groups of about equal size emerge from the data. For 42% of Swedish couples, parents engage in a trajectory of *Dual primary care* (top seven clusters). In these couples, both mothers and fathers take a substantial period of time away from work caring for their child. For another 49% of the couples, *Mother primary care* (bottom four clusters) is the main trajectory.

Second, *Dual primary care* trajectories are of two types. The dominant trajectory, employed by three out of four of the dual-caring couples (32% of all couples), was to ‘take turns’ in caregiving by providing care through *Sequential solo*-*care* (top four left-hand clusters). In this trajectory, parental care was provided through two long segments only: First, the mother cared for 8–14 months and then the father for 3–6 months. The remaining fourth of the dual-caring couples (10% of all couples) used a trajectory of ‘halving it all’ by *Alternating solo*-*care* (top three right-hand clusters). After an initial period of mother primary care, these couples shared care for a period of 5–11 months.

Third, care trajectories differed notably in the *length of parental care* provided. Despite the fixed number of paid days provided through the parental leave system, parental care was found to be of four different lengths: *very short* (about 9 months), *short* (about 13–15 months), *medium* (about 16–19 months) and *long* (about 22 months). The most common trajectory was to employ *medium parental care* lengths (50% of all couples) followed by *short parental care* (34% of all couples). Both *very short* and *long* parental care periods were uncommon (4% each).

Table 1 presents several theoretically important characteristics of each cluster of care trajectories, some of which are not easily detected in the figures. The first column provides the percentage of all couples with a particular care trajectory. The second to fifth columns present the average number of months that children in each cluster spend in each kind of primary care—mother, father or shared care—and the estimated total length of parental care. In the sixth column is found an indicator of fathers taking on the complete parental role, i.e., the percent who were solo-carers for at least 2 months.^7^ The last column provides the typical measure of care sharing in the Swedish context and elsewhere, fathers’ share of paid parental leave days (Duvander and Johansson 2012; Gíslason and Eydal 2011; Sundström and Duvander 2002).

**Table 1:** Characteristics of care strategies

		Percent	Average number of months across clusters					
			Mother primary caregiver	Father primary caregiver	Shared caregiving	Total in primary parental care	Percentage of fathers solo-caring for 2 consecutive months^a^	Fathers’ share of paid parental leave days
	**1. Dual primary care**							
	*1a. Sequential solo*-*care*							
1	Short parental care	5.0	8.3	4.4	1.2	13.9	99.1	36.3
2	Medium parental care 1	8.7	9.0	6.0	1.2	16.2	99.5	40.3
3	Medium parental care 2	11.5	11.1	5.1	1.1	17.4	100.0	34.2
4	Medium parental care 3	6.9	13.6	2.5	2.0	18.0	66.9	18.3
	Total ‘Sequential solo-care’	32.0	10.6	4.7	1.3	16.7	92.6	32.8
	*1b. Alternating solo*-*care*							
5	Short parental care	5.3	8.9	0.6	4.9	14.5	16.7	18.4
6	Medium parental care 1	2.9	7.5	2.9	8.3	18.7	51.6	33.7
7	Medium parental care 2	2.0	3.5	1.6	10.9	16.0	34.9	35.2
	Total ‘Alternating solo-care’	10.1	7.5	1.4	7.1	16.0	30.1	26.0
	Total ‘Dual primary care’	42.2	9.8	3.9	2.7	16.5	77.6	31.2
	**2. Mother primary care**							
8	Very short parental care	3.5	8.1	0.1	0.3	8.6	2.6	9.0
9	Short parental care	24.1	12.7	0.1	0.5	13.3	4.0	5.2
10	Medium parental care	17.7	16.0	0.6	1.0	17.6	15.2	6.2
11	Long parental care	3.7	18.3	0.8	2.9	21.9	19.0	9.9
	Total ‘Mother primary care’	48.9	14.0	0.3	0.9	15.2	9.0	6.2

^a^Note that the caring period is in most cases longer than 2 months if it starts or ends at any other time than at the turn of the month

Despite the considerable flexibility of the Swedish parental leave system, in which parents could shift care even by the hour, most dual-caring couples ‘take turns’ through *Sequential solo*-*care*. Three quarters of the dual-caring couples (Clusters 1–4, 32% of all couples) divide their leave this way. The caring periods are remarkably ‘pure’ as the care strategy includes hardly any shared caregiving; only about 1 month (Column 4 in Table 1) is shared between the parents throughout the entire period. Months in shared caregiving are almost as low as in the *Mother primary care* clusters, on average 1.3 months as compared to 0.9 months. In three of the four clusters employing sequential solo-care through long segments of care, more than 99% of the fathers take their leave in a solo-caring period of at least 2 months (Column 6 in Table 1). Most of the dual-caring couples do not, however, divide care perfectly equally, consistent with prior research on average leave-taking. Only parents in Cluster 2, whose long ‘turns’ are the closest in length, share paid parental leave days in the 40–60 range.

About one in four of the dual-caring couples, or about 10% of all couples, uses a trajectory of ‘halving it all’ through repeatedly *Alternating solo*-*care*. Parents in these clusters share monthly caregiving for a period of 5–11 months. This trajectory is distinct from that of *Sequential solo*-*care* through the number of months only fathers provided primary care (on average 1.4 months as compared to 4.7 months in the *Sequential solo*-*care* trajectory) and also by the low numbers of fathers solo-caring for two consecutive months (on average 30% as compared to 93%). The *Alternating solo*-*care* clusters do not differ, however, from most of the *Sequential solo*-*care* clusters when it comes to the number of paid parental leave days taken by fathers. Except for Cluster 2, in which fathers take about 40% of the paid parental leave days, the share of days taken in the two clusters is remarkably similar. Only a longitudinal measure can reveal this difference in care trajectories.

The ‘halving it all’ trajectory, stressing equal involvement as early as possible and throughout the ages, is, however, only partially supported in the Swedish context. First, although couples employing *Alternating solo*-*care* indeed share care also in the cross section, mother primary care in the first period of the child’s life remains part of the trajectory. As shown in the plots of representative children for these clusters in Fig. 1, only the last of these three clusters start to share care early on, after only one and 2 months of mother primary care. This finding is consistent with the strong Swedish norms on breastfeeding (Galtry 2003) and the consequent requirement for the mother’s presence in the first period of the child’s life. Second, 30% of couples using the *Alternating solo*-*care* trajectory also included a period of two consecutive months of father solo-caring. Rather than ‘halving it all’ throughout the child’s life, the *Alternating solo*-*care* couples use a rather mixed trajectory that may include both mother only, father only and shared primary caregiving. The *Sequential solo*-*care* trajectory may in this sense be described as a purer trajectory because it contains very little shared caregiving.

The other half of Swedish couples instead use the trajectory of *Mother primary care*. Although fathers in these couples take some paid parental leave (around 5–10%), father caring is scattered and amounts to only about 0.3 months in primary care and 0.9 months in shared primary care. We cannot know from these data the extent to which responsibility was handed over to the father. Leave could be taken together with the mother’s vacation or in connection to holidays. Although fathers in these couples may envision equal responsibilities for their children, earlier studies have shown difficulties for fathers to maintain dual-caring outside of work hours (Miller 2011). The *Mother primary care* group, however, includes some heterogeneity, for example in how 15% of fathers in Cluster 10 and 19% of fathers in Cluster 11 did solo-care for at least two consecutive months. A careful examination of these clusters (data not shown here) reveals that most of these fathers solo-cared in a period surrounded by mother primary care rather than in the pattern typical of the *Sequential solo*-*care* trajectory in which couples took turns only once. If the fathers took on full responsibility during those 2 months, these couples may be conceptually more similar to the dual-caregiving couples.

As a first exploration of the usefulness of the care trajectory as a temporal measure of care, Table 2 provides bivariate distributions of cluster membership by union status at birth and mothers’ and fathers’ educational level, employment status and income. The marginal distribution is provided at the bottom of the table, and each set of categories provides the conditional distribution of cluster membership by each characteristic. The care trajectory conceptualization will be briefly explored by examining both whether it reflects current empirical knowledge given by non-temporal measures of care and whether it captures variation which current non-temporal measures do not.

**Table 2:** Bivariate distributions of cluster membership by union status at birth and mothers’ and fathers’ educational level, employment status and income

	Freq.	%	1. Dual primary care							2. Mother primary care			
			la. Sequential solo-care				lb. Alternating solo-care						
			Short parental care (Cluster 1)	Medium parental care 1 (Cluster 2)	Medium parental care 2 (Cluster 3)	Medium parental care 3 (Cluster 4)	Short (Cluster 5)	Medium parental care 1 (Cluster 6)	Medium parental care 2 (Cluster 7)	Very short parental care (Cluster 8)	Short parental care (Cluster 9)	Medium parental care (Cluster 10)	Long parental care (Cluster 11)
Union status													
Co-residing	1137	52.7	5.0	9.9	12.5	7.3	5.9	2.8	2.3	2.4	23.4	17.9	4.0
Married	763	35.4	5.4	9.0	11.8	6.3	5.2	3.7	1.8	4.3	22.2	15.1	3.3
Out of union	258	12.0	3.5	2.7	6.2	6.6	2.7	0.8	1.2	6.2	33.0	24.0	3.5
Education mother													
Primary	258	12.0	3.5	3.9	0.8	3.1	3.5	1.2	0.8	7.4	43.0	18.2	0.8
Secondary	875	40.6	4.0	5.4	7.2	6.9	5.4	2.4	2.9	3.5	29.3	22.9	3.9
Tertiary	1025	47.5	6.2	12.8	17.9	7.8	5.7	3.7	1.6	2.5	14.9	13.1	4.2
Education father													
Primary	280	13.0	1.8	4.3	3.2	3.6	3.2	0.7	2.5	6.1	47.9	16.8	1.1
Secondary	1091	50.6	5.3	6.6	7.7	6.7	6.0	2.8	2.2	3.3	26.7	21.1	4.1
Tertiary	787	36.5	5.6	13.2	19.7	8.3	5.1	3.8	1.5	2.9	12.1	13.2	3.9
Employment status mother													
Unskilled empl.	757	35.1	5.0	5.4	7.7	7.1	5.7	2.4	2.1	2.5	28.5	24.6	4.5
Skilled empl.	828	38.4	4.8	13.8	19.9	9.5	7.4	4.0	1.5	2.2	11.8	14.3	4.4
Self-empl.	46	2.1	4.4	10.9	10.9	0.0	2.2	6.5	8.7	6.5	17.4	10.9	4.4
Not in empl.	527	24.4	5.1	5.3	3.8	2.9	1.7	1.5	2.1	6.8	37.6	13.7	1.3
Employment status father													
Unskilled empl.	856	39.7	5.5	7.4	9.5	6.0	6.4	2.5	2.7	2.7	25.6	20.2	3.6
Skilled empl.	745	34.5	5.9	13.2	20.3	9.3	5.6	4.0	1.9	2.0	9.8	14.6	4.4
Self-empl.	143	6.6	4.9	6.3	3.5	7.0	7.0	4.2	3.5	2.1	24.5	21.7	4.2
Not in empl.	414	19.2	2.2	4.4	2.7	4.4	1.7	1.2	0.2	8.5	46.6	16.4	2.2
Income mother													
1st quintile	431	20.0	4.4	3.7	4.2	2.8	1.6	1.2	2.3	7.2	38.5	12.3	0.5
2nd quintile	432	20.0	5.8	7.2	6.0	2.6	4.4	2.3	2.6	3.9	33.1	22.0	3.7
3rd quintile	431	20.0	5.6	5.1	11.4	9.7	7.2	2.3	2.3	2.1	23.7	19.7	5.8
4th quintile	432	20.0	3.0	10.9	14.8	10.7	7.2	4.2	1.4	1.9	13.4	21.8	5.8
5th quintile	432	20.0	6.0	16.7	21.1	8.6	6.0	4.4	1.4	2.6	11.8	12.5	2.6
Income father													
1st quintile	431	20.0	2.3	4.9	3.0	3.3	1.4	1.2	0.5	7.9	44.6	18.3	3.5
2nd quintile	432	20.0	7.4	7.9	7.6	5.8	5.6	3.0	2.3	2.3	27.8	18.5	2.8
3rd quintile	431	20.0	5.3	9.3	12.5	6.5	7.4	3.9	2.3	2.6	21.6	17.6	3.9
4th quintile	432	20.0	4.4	9.3	15.3	10.0	6.3	3.5	3.5	2.6	16.2	17.1	3.7
5th quintile	432	20.0	5.3	12.3	19.0	8.8	5.8	2.8	1.4	2.3	10.4	16.7	4.4
Total (frequency)			107	188	248	148	114	62	43	76	520	381	79
Total^a^ (%)			5.0	8.7	11.5	6.9	5.3	2.9	2.0	3.5	24.1	17.7	3.7

^a^Percentage distribution reflects all 17 clusters. The additional 6 clusters are shown in “Appendix”

The realization of *Mother primary care* or *Dual primary care* follows previous results for the included characteristics (e.g., Duvander and Johansson 2012; Gíslason and Eydal 2011; Sundström and Duvander 2002). The most likely trajectory for mothers and fathers with primary education, low income and unskilled employment is *Mother primary care*, while *Dual primary care* is most likely for tertiary educated, higher-income, skilled employment mothers and fathers. Previous research shows that education of both the mother and the father is a key determinant of father care (Gracia 2014), presumably through its link to gender egalitarian attitudes (Duvander 2014; Geisler and Kreyenfeld 2011). This is strongly reflected especially in care trajectories for which sharing is the greatest. Around half of tertiary educated mothers and fathers are found in clusters in which sharing of parental leave is the greatest (Clusters 1, 2, 3, 6 and 7), while only around 12% of primary educated mothers and 14% of primary educated fathers are found in these clusters. Results for the length of the parental care period follow previous research showing that mothers and fathers with higher education spend more time in child care than those with lower education (Gracia 2014), but in the Swedish context also higher income and mothers and fathers in skilled employment to larger extent realize a parental care period of medium length than do mothers and fathers with unskilled employment and lower income.

In addition to current knowledge, the care trajectory conceptualization shows that the dominant trajectory for gender egalitarian mothers and fathers, here reflected in tertiary educated mothers and fathers, is dual-caring through *Sequential solo*-*care*. In fact, the bivariate distributions show no clear pattern by education for the realization of the *Alternating solo*-*care* trajectory; tertiary educated are no more likely to realize the *Alternating solo*-*care* trajectory than are secondary educated, suggesting that the ‘taking turns’ strategy documented in previous interview studies (Eerola 2014; Farstad 2014; Roman 2014; Stefansen and Farstad 2010) may indeed be the dominant strategy that Swedish gender egalitarian mothers and fathers seek to employ. There is instead some indication that the realization of the ‘halving it all’ strategy may be driven by work-related rather than family-related characteristics. Groups with an above-average realization of the *Alternating solo*-*care* trajectories are self-employed mothers and fathers, and those with a below-average realization are mothers and fathers not in employment. Taking advantage of the uniquely flexible parental leave system may for self-employed be a way to limit the relatively higher costs of absence (Anxo and Ericson 2015). Further research including more indicators of workplace characteristics, larger numbers of mothers and fathers and full statistical models is needed to understand the differentiation between realizing a trajectory of *Alternating solo*-*care* rather than *Sequential solo*-*care*.

As a reflection of the holistic perspective of a sequence-based approach, the care trajectory conceptualization also reveals how contemporary dual-caring parents share leave to reduce mother care while, at the same time, lengthening the parental care period. There is a clear concentration of mothers more likely to be career-oriented, those with high education, high income and skilled employment, in Clusters 2 and 3. These care trajectories combine a mother primary care period shorter than the 12 months provided by the parental leave system with a parental care period of 16–17 months. Because parental care lengths of less than 12 months in Sweden (the time of availability of public child care) are rarely affordable for other than those with very high incomes, the length of these mother care periods would not be possible without father care. The very short and short mother care trajectories are instead not dominated by mothers more likely to be career-oriented but rather by those with fewer resources (primary educated, lowest income mothers and father not in employment).

## Discussion

Gendered responses to timely inflexible child care demands are increasingly recognized for their importance in reproduction of gender inequality in the family as well as in the labor market. Distinguishing ways in which couples may challenge current gender barriers is required to understand the struggles to ‘undo’ gender. Evidence from interview and observational studies led to a conceptualization of a mother–father care trajectory over the child’s early years. Two ideal-typical trajectories, ‘halving it all’ and ‘taking turns,’ were measured using a new source of couple-level longitudinal information on parents’ caregiving in contemporary Sweden.

Despite a uniquely flexible parental leave system, most Swedish dual-caring couples realized a trajectory of ‘taking turns.’ First, the mother takes leave for 8–14 months, and then the father takes leave for 3–6 months. Although the parent in paid work obviously engages in care during off-work hours (Alsarve and Boye 2012), caregiving that is associated with the absence from paid work is divided into two separate and unequal periods. These care trajectories do not fit the snapshot character of standard quantitative measures of division of childcare and can only be captured with a couple-level longitudinal measure. A quarter of dual-caring couples, however, ‘halved it all’ through repeated alternations of solo-care. Mothers and fathers in these couples alternated solo-caring for 5–11 months. Rather than a 50–50 division of care at all times, the alternating trajectory mixed shared caregiving with periods of only mother care and only father care. Bivariate associations, moreover, suggested that the realization of the alternating trajectory may be driven by work-related rather than family-related characteristics, perhaps as a way to limit costs of absence from the workplace.

Dual-caring is not equivalent to dividing care perfectly equally. While around half of Swedish couples engaged in dual-caring, only about 9% of the couples had a care trajectory that shared leave in the 40–60 range. Earlier interview studies have shown that when ‘circumstances’ such as the workplace and private economy are taken into account post-birth couples express instead the importance of substantial rather than equal father care (Alsarve and Boye 2012; Roman 2014; Roman and Peterson 2011). These results show that this idea may be rather widespread among Swedish dual-caregiving couples.

This study documents a considerable diversity in the caring experiences of children growing up in Sweden today. We could perhaps talk about two different caring regimes. Around half of the children grow up with dual-caring parents and may be given the possibility to develop a relationship with their fathers that is not only closer but also of a different character than that with a ‘traditional’ father (Almqvist and Duvander 2014; Haas and Hwang 2008). The other half grows up with their mothers providing the bulk of primary care, a regime that may be described as a failure of the gender equality orientation of the Swedish welfare state (Orloff 2009). For these families, there is a large gap between the well-documented ‘nurturing father’ discourse (Johansson and Klinth 2008) and real-life practices, or varying understandings of what it takes to become an ‘involved father.’

Although not the aim of the study, the results also revealed great diversity in how long Swedish children are in parental care. The current study showed clearly that care trajectories of dual-caring as well as mother caring couples substantially deviated from the number of days provided through the parental leave system. As seen by the dominance of leave lengths around 16–18 months rather than the 13 months that would be provided with earnings replacement, most parents seek to delay the introduction of non-parental care. Previous evidence on length of total parental care is scarce. Duvander and Viklund (2014) have estimated individual leave lengths including unpaid leave, but do not provide information on total parental care. The ways in which Swedish couples extend their leave beyond the 380 paid days provided through the parental leave system are an important topic, especially for international comparisons of leave systems (Duvander and Viklund 2014).

Theory and research stressing early involvement suggest a link between timing of father primary care and the overall sharing of care. For the dual-caring couples taking turns as primary caregivers, we cannot tell from these data whether timing of father primary care is a result of decisions about overall sharing and total parental care length or a wish for full engagement at a particular stage in the child’s life. For example, couples may have a preference for equal sharing and a total 18 months in parental care that produce the timing of father care. It may also be that timing of father care is paramount in the decision process, perhaps due to a preference for a long period of breastfeeding, and leads to variations in total parental care length. These issues cannot be resolved with only parental leave data.

Sweden provides an excellent case for understanding temporality of dual-caring, both by substantial numbers of fathers who allocate time from paid work to child care and by providing a parental leave system that allows for care trajectories ranging from ‘halving it all’ on a daily or weekly basis to ‘taking turns’ of around 6 months to 1 year each. We may expect a temporal conceptualization of care to become more important also outside of Sweden as the new discourse of engaged fathers may begin to include the allocation of time from paid work to child care. These results suggest that current cross-sectional measures of time use may fail to detect emerging trends in dual-caring as these arise in other contexts.

## Figures and Tables

**Fig. 3:**
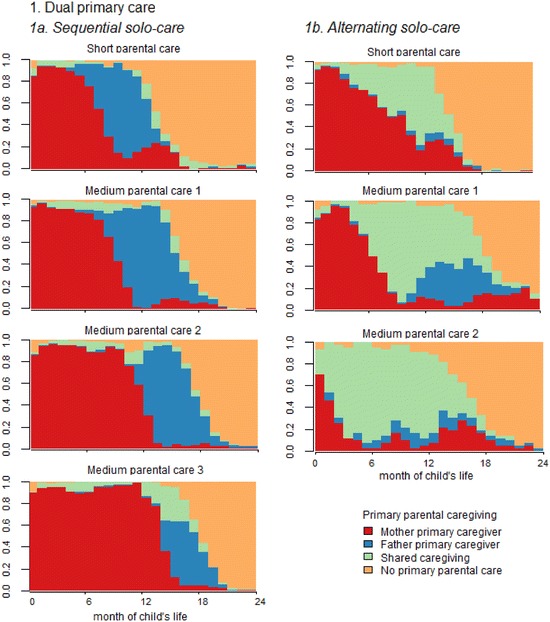

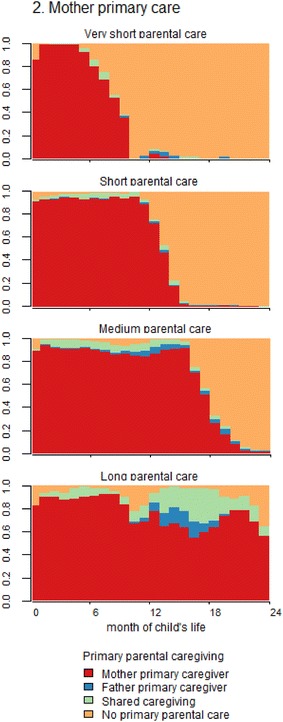

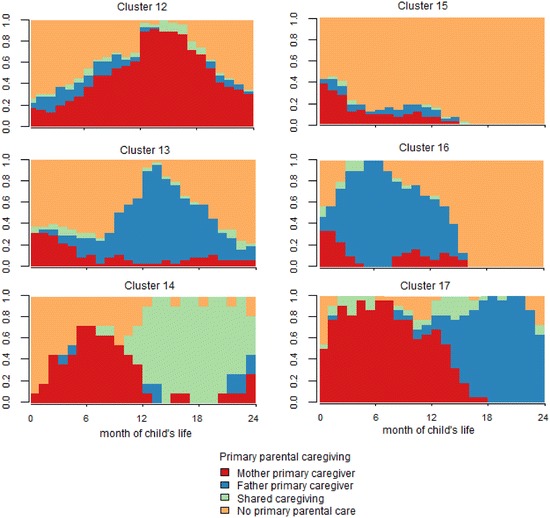
State distribution plots of care trajectory clusters as generated through sequence and cluster analysis

## Appendix

See Figs. 2, 3 and Tables 3, 4, 5.

**Table 3:** Local maxima for the average silhouette width and point biserial correlation for cluster solutions 1–20 (Studer 2013)

	1st solution	Stat	2nd solution	Stat	3rd solution	Stat	4th solution	Stat
Point biserial correlation	17	0.41	16	0.41	15	0.41	14	0.40
Average silhouette width	2	0.23	16	0.19	15	0.19	14	0.19

**Table 4:** Characteristics of care trajectory Clusters 12–17

Cluster number	Freq.	%	Average number of months across clusters					
			Mother primary caregiver	Father primary caregiver	Shared caregiving	Total in primary parental care	Percentage of fathers solo-caring for 2 consecutive months^a^	Fathers’ share of paid parental leave days
12	45	2.1	12.0	1.7	1.0	14.7	32.6	13.9
13	38	1.8	2.4	8.5	0.8	11.7	100.0	78.4
14	11	0.5	6.5	1.0	10.5	18.0	27.3	34.6
15	46	2.1	1.9	0.9	0.2	3.0	22.2	32.3
16	30	1.4	1.9	9.3	0.5	11.6	100.0	80.4
17	22	1.0	11.3	9.0	1.8	22.2	100.0	40.2

^a^Note that the caring period is in most cases longer than 2 months if it starts or ends at any other time than at the turn of the month

**Table 5:** Bivariate distributions of cluster membership 12–17 by union status at birth and mothers’ and fathers’ educational level, employment status and income

	Cluster 12	Cluster 13	Cluster 14	Cluster 15	Cluster 16	Cluster 17
Union status						
Co-residing	1.4	1.1	0.4	1.4	1.4	0.9
Married	3.0	2.8	0.5	3.0	1.3	1.3
Out of union	2.7	1.6	0.8	2.3	1.6	0.8
Education mother						
Primary	3.5	1.6	0.0	3.5	4.7	0.8
Secondary	1.8	0.7	0.2	1.7	1.3	0.7
Tertiary	2.1	2.7	0.9	2.1	0.7	1.4
Education father						
Primary	3.2	0.0	0.0	2.9	1.8	1.1
Secondary	1.7	1.0	0.6	1.7	1.9	0.8
Tertiary	2.4	3.4	0.6	2.4	0.5	1.3
Employment status mother						
Unskilled empl.	1.6	0.7	0.0	0.8	0.8	0.7
Skilled empl.	1.5	1.6	1.2	0.5	0.1	1.7
Self-empl.	4.4	2.2	0.0	4.4	4.4	2.2
Not in empl.	3.8	3.6	0.2	6.3	4.0	0.4
Employment status father						
Unskilled empl.	1.6	0.9	0.7	2.2	1.8	0.8
Skilled empl.	1.9	3.2	0.7	1.3	0.5	1.3
Self-empl.	4.9	0.7	0.0	0.7	2.8	2.1
Not in empl.	2.7	1.2	0.0	3.6	1.7	0.5
Income mother						
1st quintile	4.4	3.9	0.2	7.2	4.9	0.7
2nd quintile	1.2	1.9	0.0	1.6	1.6	0.2
3rd quintile	1.4	0.9	0.9	0.2	0.2	1.4
4th quintile	1.6	0.5	1.2	0.7	0.2	0.9
5th quintile	2.1	1.6	0.2	0.7	0.0	1.9
Income father						
1st quintile	2.6	0.9	0.0	3.3	2.1	0.5
2nd quintile	2.1	1.2	0.5	2.1	2.1	1.2
3rd quintile	1.6	0.7	0.5	1.6	1.4	1.2
4th quintile	2.3	2.3	1.2	0.7	1.2	0.7
5th quintile	2.1	3.7	0.5	2.8	0.2	1.6
Total (frequency)	46	38	11	45	30	22
Total (%)	2.1	1.8	0.5	2.1	1.4	1.0

Percentage distribution reflects all 17 clusters

## Footnotes

1 All forms of salary and wages are converted to a daily rate for 365 days per year, and that is the amount that is paid for each day claimed, including days that are not normally worked.

2 Income-related benefits may be lowered if parents do not take five paid days per week in the period after the child turns one (SSIA 2011a). If this rule is followed the maximum length of job-protected leave is 12 + 14 months.

3 The dataset includes no information on other parental leave benefits available in the Swedish parental leave system. The most important of these are the 10 days of temporary parental leave benefits that could be claimed by the parent not on parental leave in any of the 60 days following the birth of the child. About 74% of fathers (or in a few cases other secondary parents) used this benefit in 2009 (SSIA 2011a), so data including only parental leave neglect that most parents share care for at least 2 weeks in the first months.

4 All children born in 2009 are available in the dataset, but because the calculation of distances in optimal matching is both time- and memory-intensive (Gabadinho et al. 2010), analyses of the full population were not feasible with available computing resources. A number of random samples were drawn that all generated the same overall results. The full dataset includes children born in 1994 and forward, so ‘first-born’ are those in which none of the parents had another child in this period. Parents with other children older than 14 years old are thus included in the ‘first-born’ sample. ‘First-born’ rather than all children are selected so as to eliminate possibilities of parental leave still available for older siblings.

5 Annual income in 2007 is used because income in 2008 may be affected by expecting a child in early 2009. Additional analyses of associations between cluster membership and income in 2008, however, provided the same overall results.

6 The analysis presented does not consider gradations (e.g., 40–60, 30–70, etc.) of sharing. These specifications were all tested, but provided no better representation of the underlying behavior.

7 Note that by assigning primary caring by each month of the child’s life, a solo-caring period of 2 months is for most couples longer if it starts or ends at any other time than at the turn of the month.
